# Effect of Upper Robot‐Assisted Training on Upper Limb Motor, Daily Life Activities, and Muscular Tone in Patients With Stroke: A Systematic Review and Meta‐Analysis

**DOI:** 10.1002/brb3.70117

**Published:** 2024-10-31

**Authors:** Tingting Su, Mengting Wang, Zhouyang Chen, Liang Feng

**Affiliations:** ^1^ Department of Rehabilitation Medicine Tongxiang First People's Hospital Tongxiang Zhejiang China

**Keywords:** meta‐analysis, rehabilitation, robotics, stroke, systematic review, upper limb

## Abstract

**Background:**

Upper limb rehabilitation robot is a relatively new technology, but its effectiveness remains debatable due to the inconsistent results of clinical trials. This article intends to assess how upper limb rehabilitation robots help the functional recovery of stroke patients.

**Methods:**

PubMed, Embase, Cochrane Library, and Web of Science databases were searched for eligible studies to explore the effect of upper limb rehabilitation robots on upper limb motor function, muscle tone, and daily living activities.

**Results:**

Eighteen trials with 573 stroke patients met the inclusion criteria. The results showed that compared to conventional rehabilitation training, patients who received upper limb robotic therapy (RT) had significantly improved Fugl‐Meyer Upper Extremity Motor Assessment (FMA‐UE) scores (weighted mean differences [WMD]: 5.27, 95% confidence intervals [CI]: 3.36, 7.17), Action Research Arm Test (ARAT) scores (WMD: 4.07, 95% CI: −4.14, 12.28), Modified Barthel Index (MBI) scores (WMD: 9.55, 95% CI: 6.37, 12.73), and modified Ashworth Scale (MAS) scores (WMD: −0.28, 95% CI: −0.50, 0.06), with no significant heterogeneity.

**Conclusions:**

Upper limb robot–assisted training is superior to conventional training in terms of improving upper limb motor impairment, ability to perform daily living activities, and muscle tone recovery, which supports the application of robots in clinical practice.

## Graphical abstract

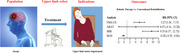

## 1 Introduction

Stroke is a cerebrovascular disease. According to the World Health Organization, over half of stroke patients have varied degrees of upper limb motor impairment, which profoundly affects their independent living ability (Lindsay et al. 2019; Yue et al. 2019).

However, about 85% of stroke patients develop upper limb dysfunction, and even within 3–6 months after stroke, 55%–75% of patients still have difficulty recovering upper limb function (Kwakkel et al. 2003). The delicacy of upper limb function determines its slower recovery than that of lower limbs, which seriously affects patients’ daily self‐care.

Many rehabilitation therapies are currently available for upper limb function recovery in stroke patients with hemiplegia, such as motor relearning, constraint‐induced therapy, neurodevelopmental therapy, and occupational therapy (Saposnik et al. 2016). However, most conventional rehabilitation (CR) therapies have some disadvantages, including a single‐training method, dependence on therapist assistance, poor interest, poor patient compliance, and difficulty in objectively quantifying the training intensity (Toh et al. 2023).

Owing to stroke patients’ growing expectations for functional recovery and the advancement of rehabilitation technology, upper limb rehabilitation robots have emerged as a novel rehabilitation approach (Rahman et al. 2023). Robots designed to assist in or replace certain human upper limb functions are known as upper limb rehabilitation robots. These mechanical devices can carry out tasks automatically and help patients implement high‐intensity, targeted, and repetitive rehabilitation training. Compared with CR, rehabilitation robots have significant advantages in promoting functional recovery of stroke patients (Chockalingam et al. 2022; Pournajaf et al. 2023).

Although there is literature suggesting that robotic therapy (RT) is superior to CR, there is no conclusive evidence that RT is more suitable for stroke patients than CR. A meta‐analysis showed no significant difference in basic daily living ability and muscle tone between RT and CR (Bertani et al. 2017).

This review aimed to evaluate the effectiveness of robot‐assisted upper limb training compared with conventional interventions in improving upper limb motor function and daily living ability in stroke patients. At present, there are many types of upper limb rehabilitation robots. The different functions of exoskeleton and end‐effector devices have also attracted much attention.

## 2 Methods

This systematic review and meta‐analysis was conducted following the Preferred Reporting Items for Systematic Reviews and Meta‐Analyses (PRISMA) statement. The study was registered in PROSPERO (CRD42023464736).

### 2.1 Search Strategy

All published articles were searched on PubMed, Embase, Cochrane Library, and Web of Science up to July 17, 2023 using the following MeSH keywords: stroke, robotics, and rehabilitation. The detailed search strategies are listed in Appendix S1.

### 2.2 Study Selection

The inclusion criteria for relevant studies were designed according to the study population, intervention, comparison, outcome, and study design. Inclusion criteria were as follows: (1) study population: stroke patients with upper limb motor impairment (no restrictions on age, gender, or stage of stroke); (2) intervention: upper limb robot rehabilitation training; (3) comparison: CR; (4) outcome: Fugl‐Meyer Upper Extremity Motor Assessment (FMA‐UE), Action Research Arm Test (ARAT), Modified Barthel Index (MBI), and Modified Ashworth Scale (MAS); and (5) study design: randomized controlled trial (RCT). Exclusion criteria included: (1) duplicates; (2) case reports, letters, comments, editorials, guidelines, notes, or reports; (3) reviews and/or meta‐analyses; (4) experimental or animal studies; (5) non‐stroke patients with movement disorders; (6) no robotic rehabilitation; and (7) literature with abnormal or missing data. There were no restrictions on the language and year of publication.

### 2.3 Data Extraction

The literature was screened by two researchers tingting su (TTS), mengting wang (MTW) independently. The full text was reviewed, and relevant data were extracted and cross‐checked. Any disagreement in study selection was tackled by discussion with a third researcher zhouyang chen (ZYC).

After initial retrieval, the titles and abstracts of included studies were read. According to the selection criteria, we excluded all ineligible studies. The full text of eligible studies was reviewed for further selection. The following variables were extracted: outcome measure, recovery phase, training duration, training length, and type of robotic device.

### 2.4 Study Quality

The quality of the included RCTs was independently evaluated by two reviewers using the Revised Cochrane Risk of Bias tool (RoB2) (Sterne et al. 2019). The RoB2 consists of seven domains: randomization process, allocation concealment, blinding of participants and personnel, blinding of outcome measurement, incomplete outcome data, selective reporting of outcomes, and other biases. There were three answers for each domain: “yes” (low risk), “no” (high risk), or “uncertain” (unclear risk). The quality of the RCTs was evaluated according to these domains to determine the overall risk of bias.

### 2.5 Statistical Analyses

STATA 15.0 (Stata Corp, College Station, Texas, USA) and RStudio 4.3.1 (R Foundation for Statistical Computing, Vienna, Austria) were used for statistical analyses. A fixed‐effect model was employed. Continuous outcomes were depicted as weighted mean differences (WMDs) with 95% confidence intervals (CI). The Cochran's *Q* test and *I* ^2^ statistics were utilized to evaluate the heterogeneity, with *p* <  0.1 and/or *I* ^2^ >  50% indicating marked heterogeneity. Publication bias was assessed with the Egger test, with *p* < 0.05 indicating statistically significant publication bias (Egger et al. 1997). Meta‐regression analyses and sensitivity analyses were used to determine the sources of heterogeneity (Thompson and Sharp 1999). Stroke latency (≤ 6 vs. > 6 months), training length (≤ 40 vs. > 40 min), total training duration (≤ 4 vs. > 4 weeks), and type of robot (end‐effector and exoskeleton) were included in meta‐regression analyses. If the number of included RCTs were ≥ 2, subgroup analyses would be conducted according to the above‐mentioned covariates, with *p* <  0.10 representing significant interactions.

## 3 Results

### 3.1 Study Characteristics

**FIGURE 1:**
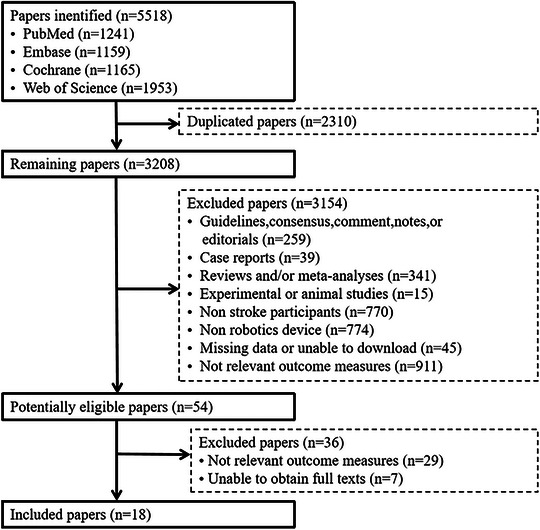
Flow chart of study selection.

Initially, 5518 papers were identified, and 18 papers (Changcheng et al. 2018; Chao et al. 2016; Chen et al. 2021; Chinembiri et al. 2021; Coskunsu et al. 2022; Frisoli et al. 2022; Guo et al. 2022; Hsu et al. 2022; Jiang et al. 2021; Kim et al. 2021; Lee, Lee, and Lee 2018; Lee et al. 2021; Ma et al. 2022; Ranzani et al. 2020; Şenocak et al. 2023; Singh et al. 2021; Taravati et al. 2022; Yuan et al. 2023) were finally included. The literature screening flow chart is shown in Figure 1.

### 3.2 Study Characteristics

The 18 included RCTs were conducted in Italy, Turkey, India, Korea, Switzerland, and China. A total of 573 patients were enrolled, with an average age of 52 years in the RT group and 56 years in the CR group. Stroke latency was categorized as ≤ 6 and > 6 months. The total training cycle ranged from 2 to 9 weeks. The duration of each training ranged from 20 to 60 min, and the frequency ranged from 2 to 6 days each week. The type of robot included end‐effector and exoskeleton. No adverse event was reported in any included studies. The basic characteristics are shown in Table 1.

**TABLE 1:** Basic characteristics of included studies.

Study (year)	Design	Location	Intervention (*n*)		Recovery phase (months)	Duration (weeks)	Training length (per session) (min)	Type of robotic	Outcome measures
			Treatment	Control					
Yuan et al. (2023)	RCT	China	RT (23)	CR (23)	≤ 6	3	60	End‐effector	FMA‐UE, MBI
Şenocak et al. (2023)	RCT	Turkey	RT (19)	CR (22)	≤ 6	6	60	End‐effector	FMA‐UE
Taravati et al. (2022)	RCT	Turkey	RT (17)	CR (20)	> 6	4	30	End‐effector	FMA‐UE, MAS
Ma et al. (2022)	RCT	China	RT (10)	CR (9)	≤ 6	4	90	Exoskeleton	FMA‐UE, ARAT
Hsu et al. (2022)	RCT	China	RT (17)	CR (15)	> 6	9	40	Exoskeleton	FMA‐UE, MAS
Guo et al. (2022)	RCT	China	RT (10)	CR (10)	> 6	2	60	Exoskeleton	FMA‐UE, MAS
Frisoli et al. (2022)	RCT	Italy	RT (11)	CR (11)	> 6	6	45	End‐effector	FMA‐UE
Coskunsu et al. (2022)	RCT	Turkey	RT (11)	CR (19)	≤ 6	3	60	End‐effector	FMA‐UE, ARAT
Singh et al. (2021)	RCT	India	RT (12)	CR (11)	> 6	4	45	Exoskeleton	FMA‐UE, MBI, MAS
Lee et al. (2021)	RCT	China	RT (14)	CR (10)	> 6	6	60	End‐effector	FMA‐UE, MBI
Kim et al. (2021)	RCT	Korea	RT (23)	CR (24)	> 6	4	30	End‐effector	FMA‐UE, MAS
Jiang et al. (2021)	RCT	China	RT (23)	CR (22)	≤ 6	2	30	Exoskeleton	FMA‐UE, MBI
Chinembiri et al. (2021)	RCT	China	RT (20)	CR (25)	≤ 6/> 6	6	50	End‐effector	FMA‐UE, MBI
Chen et al. (2021)	RCT	China	RT (10)	CR (10)	≤ 6	4	45	Exoskeleton	FMA‐UE, ARAT, MBI
Ranzani et al. (2020)	RCT	Switzerland	RT (14)	CR (13)	≤ 6	4	45	End‐effector	FMA‐UE
Sun (2018)	RCT	China	RT (38)	CR (32)	≤ 6	4	30	Exoskeleton	FMA‐UE, MBI
Lee, Lee, and Lee (2018)	RCT	Korea	RT (15)	CR (15)	> 6	8	30	Exoskeleton	FMA‐UE, MBI
Zhang (2016)	RCT	China	RT (6)	CR (6)	> 6	4	20	End‐effector	FMA‐UE, MAS

Abbreviations: ARAT, Action Research Arm Test; CR, conventional rehabilitation; MAS, modified Ashworth Scale; MBI, Modified Barthel Index; RCT, randomized controlled trial; RT, robotic therapy.

### 3.3 Methodological Quality of Included RTCs

**FIGURE 2:**
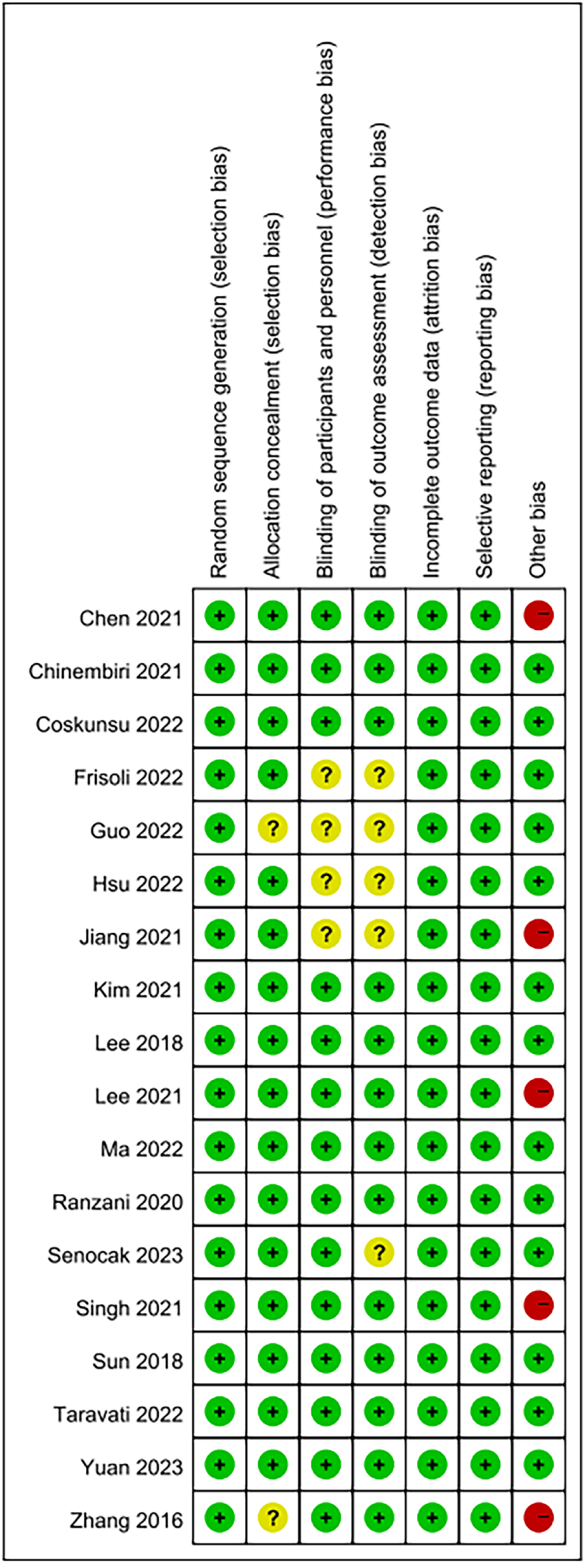
Risk of bias for 10 studies (green; low risk, yellow; unclear, red; high risk).

According to RoB 2.0, nine RCTs were rated as having a low risk of bias, four RTCs as having some concerns, whereas the other five RTCs were rated as having a high risk of bias. The detailed risk of bias is shown in Figure 2.

### 3.4 Primary Outcomes

#### 3.4.1 FMA‐UE Scores

**FIGURE 3:**
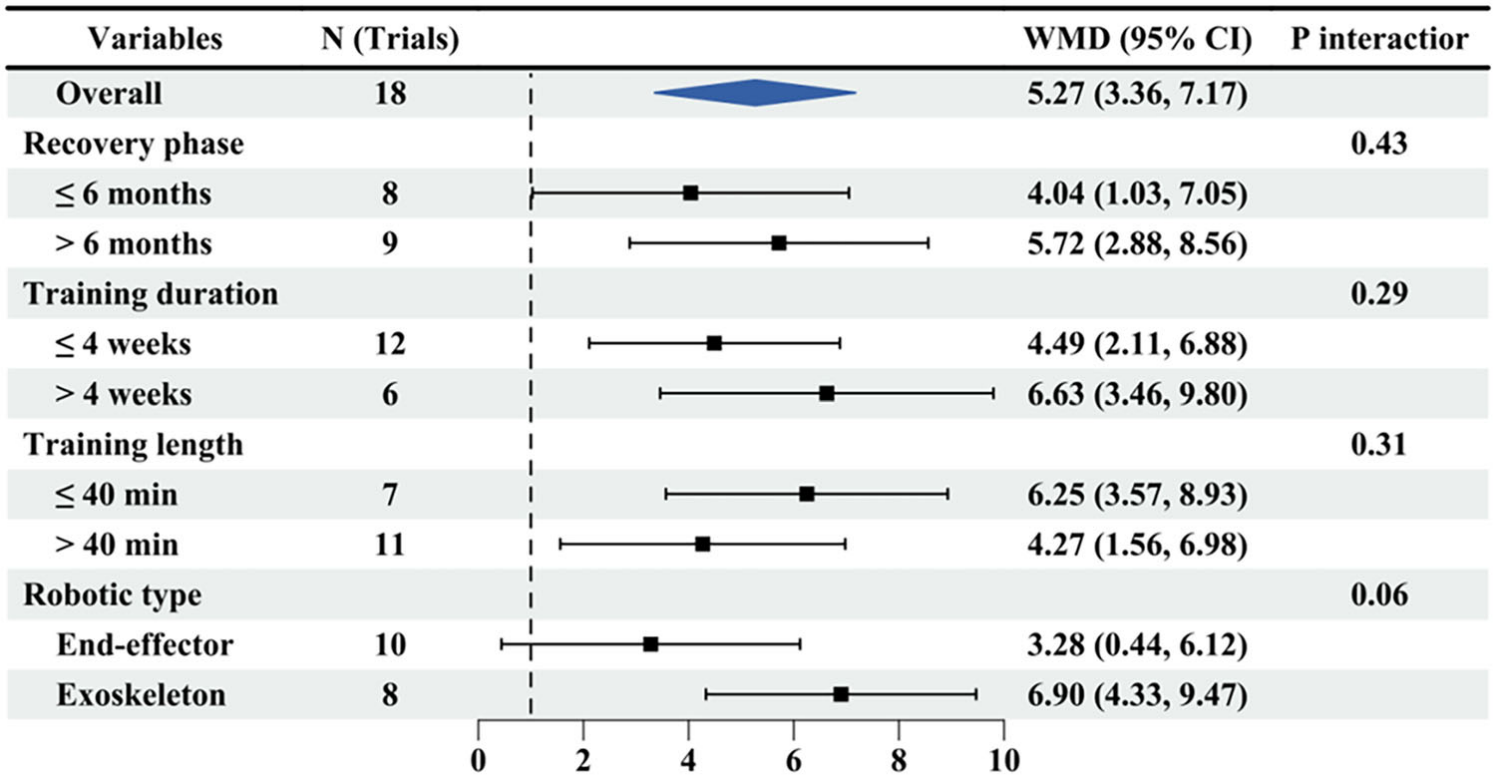
Subgroup results of RT versus CR on FIM‐UE. CR, conventional rehabilitation; RT, robotic therapy.

The FMA‐UE scale was used to measure upper limb function in 18 papers (Figure 3). Patients who received upper limb RT had significantly improved FMA‐UE scores after the intervention (WMD: 5.27, 95% CI: 3.36, 7.17), with no significant heterogeneity (*I* ^2^ = 0%, *p* = 0.77). Subgroup analyses revealed that in patients with upper limb RT, its efficacy was better in the chronic phase (latency > 6 months) (WMD: 5.72, 95% CI: 2.88, 8.56) than in the subacute phase (latency ≤ 6 months) (WMD: 4.04, 95% CI: 1.03, 7.05), with no significant difference (*p* = 0.43). The efficacy of the intervention for > 4 weeks (WMD: 6.63, 95% CI: 3.46, 9.80) was better than that for ≤ 4 weeks (WMD: 4.49, 95% CI: 2.11, 6.88), with no notable difference (*p* = 0.29). Treatment effects of upper limb RT were better at intervention duration of > 40 min (WMD: 4.27, 95% CI: 1.56, 6.98) than that of ≤ 40 min (WMD: 6.25, 95% CI: 3.57, 8.93), with no evident difference (*p* = 0.31). Exoskeleton (WMD: 6.90, 95% CI: 4.33, 9.47) had a better effect on upper limb motor function than the end‐effector (WMD: 3.28, 95% CI: 0.44, 6.12), and the difference was statistically significant (*p* = 0.06) (Figure 3). There was no significant publication bias (*p* = 0.768).

### 3.5 Secondary Outcomes

#### 3.5.1 ARAT Scores

**FIGURE 4:**
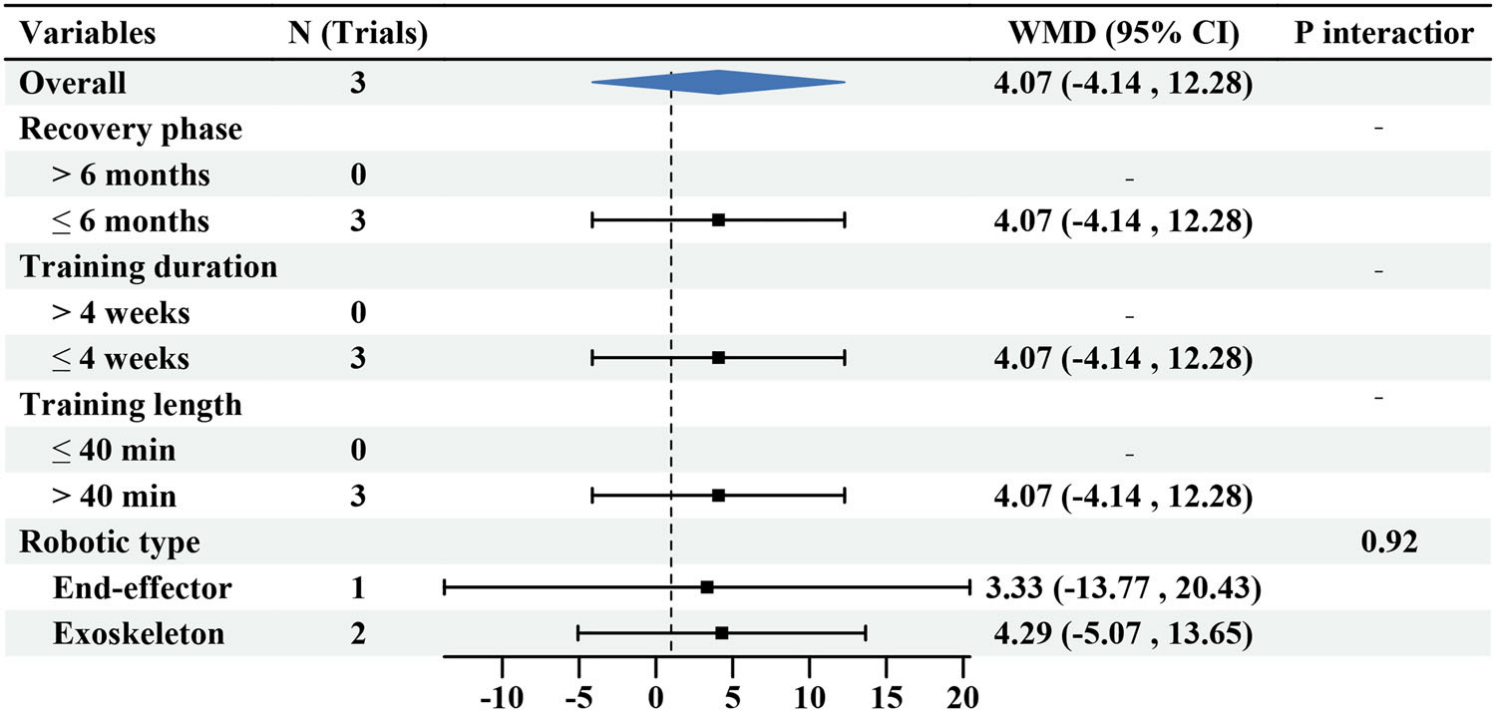
Subgroup results of RT versus CR on ARAT. ARAT, Action Research Arm Test; CR, conventional rehabilitation; RT, robotic therapy.

Patients who received upper limb RT had significantly improved ARAT scores after the intervention (WMD: 4.07, 95% CI: −4.14, 12.28) (Figure 4), with no significant heterogeneity (*I* ^2^ = 0%, *p* = 0.91). Subgroup analyses found that exoskeleton (WMD: 4.29, 95% CI: −5.07, 13.65) had a better effect on upper limb motor function than the end‐effector (WMD: 3.33, 95% CI: −13.77, 20.43), but there was no marked difference (*p* = 0.92).

#### 3.5.2 MBI Scores

**FIGURE 5:**
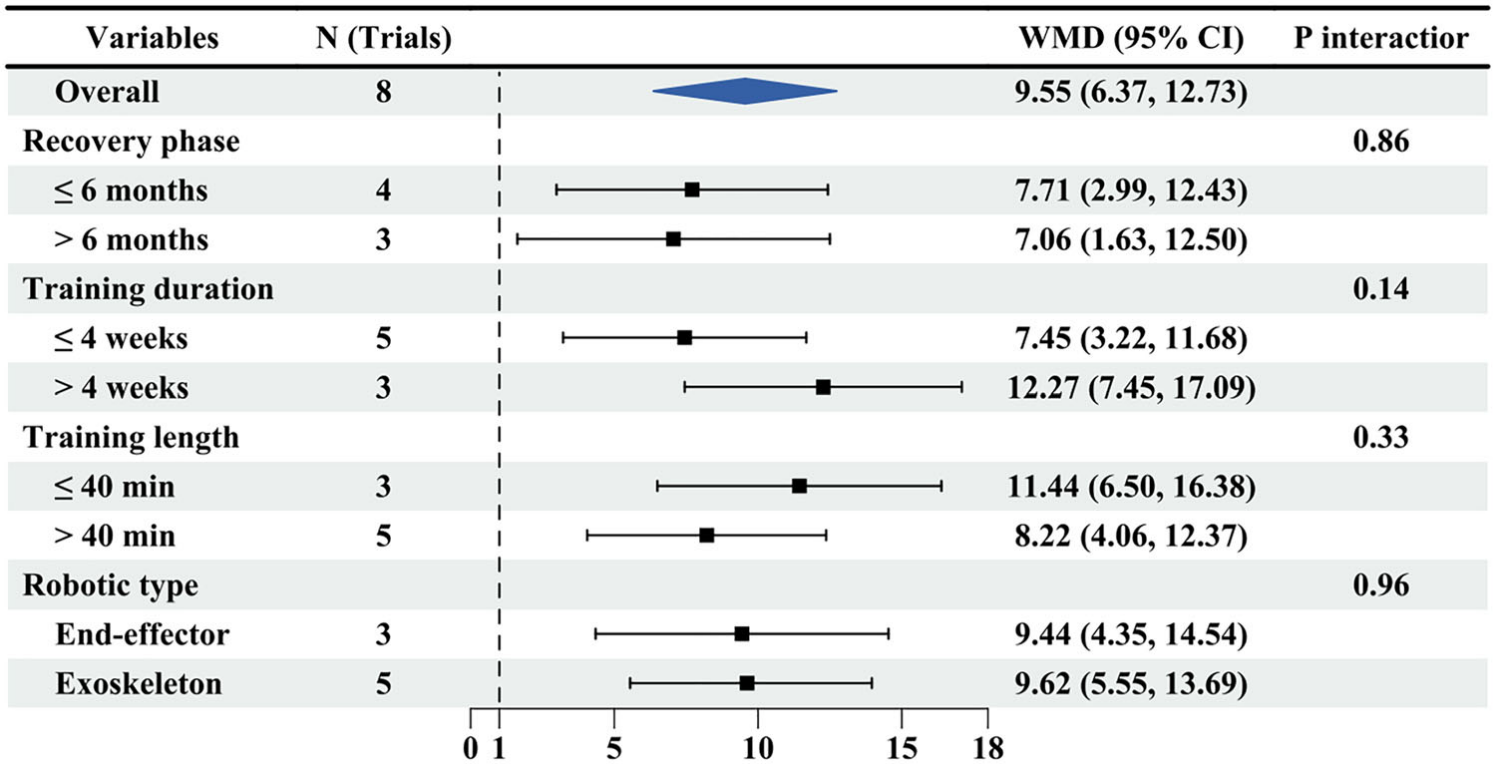
Subgroup results of RT versus CR on MBI. MBI, Modified Barthel Index; CR, conventional rehabilitation; RT, robotic therapy.

The pooled WMD of MBI scores from eight trials was examined. Patients who received upper limb RT had significantly improved MBI scores after the intervention (WMD: 9.55, 95% CI: 6.37, 12.73) (Figure 5), with no significant heterogeneity (*I* ^2^ = 50%, *p* = 0.05). As subgroup analyses revealed, in patients with upper limb RT, the efficacy was better in the subacute phase (WMD: 7.71, 95% CI: 2.99, 12.43, *I* ^2^ = 40%) than in the chronic phase (WMD: 7.06, 95% CI: 1.63, 12.50), but there was no significant difference (*p* = 0.86). The efficacy of the intervention for > 4 weeks (WMD: 12.27, 95% CI: 7.45, 17.09) was better than that for ≤ 4 weeks (WMD: 7.45, 95% CI: 3.22, 11.68), with no significant difference (*p* = 0.14). The efficacy was better with treatment duration > 40 min (WMD: 8.22, 95% CI: 4.06, 12.37) than that of ≤ 40 min (WMD: 11.44, 95% CI: 6.50, 16.38), with no evident difference (*p* = 0.33). Exoskeleton (WMD: 9.62, 95% CI: 5.55, 13.69) had a better effect on upper limb motor function than the end‐effector (WMD: 9.44, 95% CI: 4.35, 14.54), with no marked difference (*p* = 0.96). There was no significant publication bias (*p* = 0.85).

#### 3.5.3 MAS Scores

**FIGURE 6:**
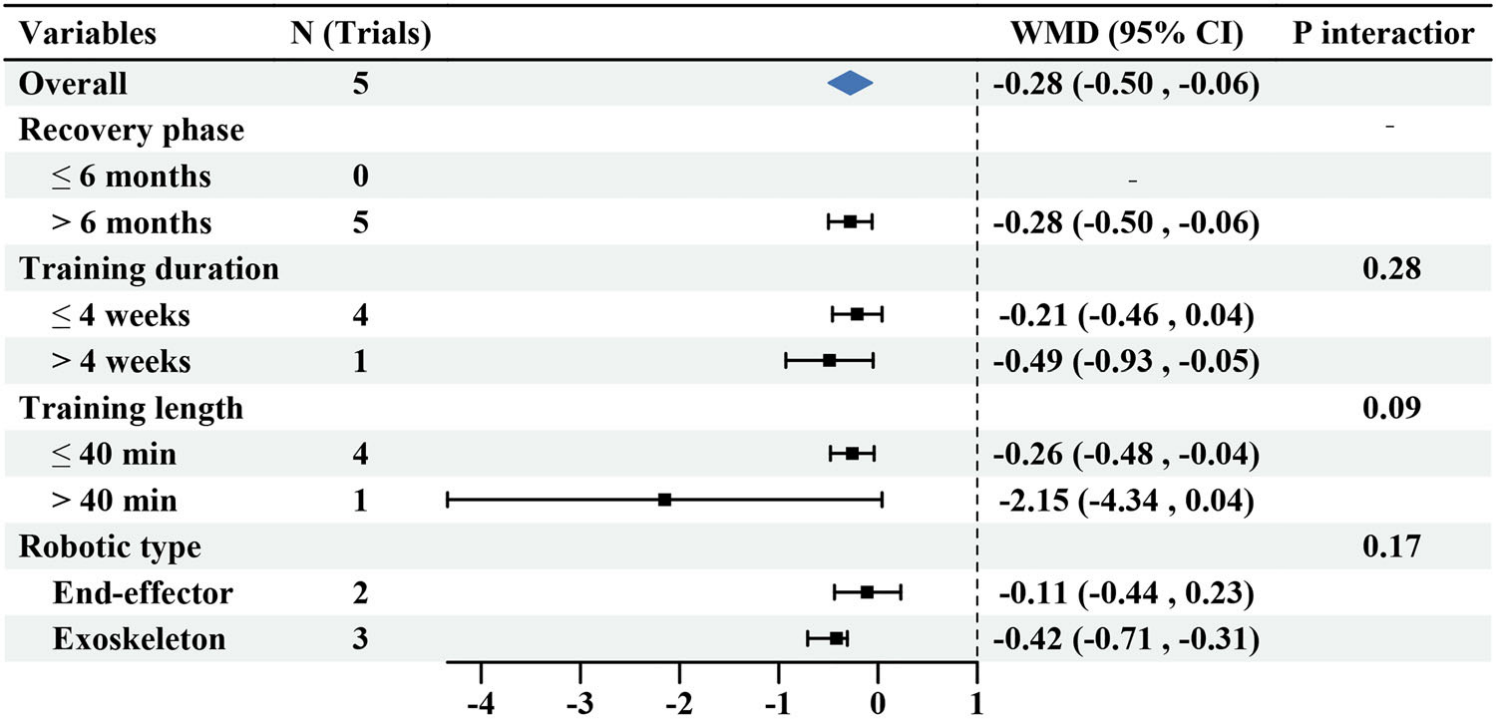
Subgroup results of RT versus CR on MAS. MAS, modified Ashworth Scale; CR, conventional rehabilitation; RT, robotic therapy.

The pooled WMD of MAS scores from five trials was examined. Patients who received upper limb RT had significantly improved MAS scores after the intervention (WMD: −0.28, 95% CI: −0.50, 0.06) (Figure 6), with no significant heterogeneity (*I* ^2^ = 18%, *p* = 0.30). Subgroup analyses revealed that the efficacy of the intervention for > 4 weeks (WMD: −0.49, 95% CI: −0.93, −0.05) was better than that for ≤ 4 weeks (WMD: −0.21, 95% CI: −0.46, 0.04), but there was no marked difference (*p* = 0.28). There were significant differences between treatment durations of ≤ 40 min (WMD: −0.26, 95% CI: −0.48, −0.04) and > 40 min (WMD: −2.15, 95% CI: −4.34, 0.04; *p* = 0.09). Exoskeleton (WMD: −0.42, 95% CI: −0.71, −0.31) had a better effect on upper limb motor function than the end‐effector (WMD: −0.11, 95% CI: −0.44, 0.23), without significant difference (*p* = 0.17).

## 4 Discussion

Previous meta‐analyses have confirmed the positive effect of robot‐assisted training on upper limb motor recovery, but the effects on muscle tone and daily living activities are inconsistent (Bertani et al. 2017; Chen et al. 2020; Lee, Saragih, and Batubara 2023). The results of this study showed that the FMA‐UE, ARAT, MBI, and MAS scores of the RT group were significantly better than those of the CT group, suggesting that the robot‐assisted training on upper limb rehabilitation can improve the motor function of the upper limb and hand, enhance the ability of daily living, and reduce muscle tension in stroke patients. In the subgroup analysis, our results uncovered that the exoskeleton robot could significantly improve the motor function of the hemiplegic upper limb compared with the end‐effector robot. After stroke, the recovery of upper limb function mainly depends on the recovery, adaptation, and relearning of the nervous system. The meta‐analysis results of this study showed that the FMA‐UA score of the experimental group was higher than that of the control group after intervention. It is possible that upper limb robot‐assisted rehabilitation training can promote brain remodeling through high‐intensity, high‐repetition, and high‐precision training to improve the upper limb motor function of stroke patients (Chien et al. 2020). Furthermore, the upper limb rehabilitation robot, by delivering feedback, can guide the patient into a preset mode through initiative and movement, thus better assisting the patient in achieving fatigue‐free and continuous upper limb movement. On the basis of the brain's plasticity, the training difficulty is gradually adjusted, and the brain is stimulated through repetitive movements to make up for the missing function of the brain (Dehem et al. 2019).

ARAT mainly assesses the ability to grip and move multiple objects within a limited workspace, thus evaluating the motor performance quality related to compensatory shoulder and arm movements during griping (Lang et al. 2008). The meta‐analysis noted higher ARAT scores in the RT group than that in the CT group after the intervention. This result may be attributed to the advantage of auxiliary upper limb robot training in driving single or multiple joints of the upper limb to perform passive and active activities, enhancing the muscle strength of the affected limb, improving the coordination between joints, and promoting the recovery of fine motor abilities such as grip, release, and operation. Bertani et al. (2017) showed that robotic rehabilitation did not significantly improve muscle tone in patients’ daily living activities. However, the results of the present study showed significant improvements in quality of life and muscle tone with the rehabilitation robot. It is well known that abnormal muscle tone and joint stiffness impede motor performance and lead to reduced or even loss of motor activity. Conversely, when muscle tone is reduced, active exercise will facilitate functional retraining (Hellsten et al. 2008). At the same intervention time, due to its high repetitive training, the upper limb rehabilitation robot can more effectively relieve the upper limb flexor spasticity and improve the motion range of the upper limb joint in stroke patients by stretching the spastic muscles of the shoulder and wrist and promoting the contraction of the antagonistic muscles. A temporary enhancement in functional status can be seen as a therapeutic “window of opportunity,” during which the patient can use the residual function to achieve better motor outcomes. In terms of daily living activities, situational task‐oriented training can encourage the patients to master specific activity skills and continuously improve their daily living ability (Zengin‐Metli et al. 2018). In addition, the improved upper limb motor function in stroke patients can improve daily living ability.

In terms of subgroup analysis, the subgroup analysis of FMA‐UE revealed that the exoskeleton robot yielded more significant improvements. Exoskeleton robot is designed based on bionic principles and motion mechanisms of the human upper limb. Its joints are aligned with the joint axis of the patient's upper limb, ensuring a close connection for those with upper limb dysfunction. Exoskeleton robots provide a greater range of motion than end‐effector robots, thus ensuring optimal control of arm and wrist movements (Calabrò et al. 2021). In addition, the exoskeleton robot is advantageous for establishing correct sensory‐motor pathways and facilitating the remodeling of the nervous system. Repeated task‐oriented training by wearing a high‐precision exoskeleton can enhance proprioceptive stimulation, facilitate memory formation, and refine movement patterns, thereby remodeling the nervous system and cerebral cortex and ultimately promoting the recovery of upper limb function (Calabrò et al. 2016; Chen et al. 2023). Our subgroup analysis found no significant difference in the stroke latency, training duration, and training cycle of FMA‐UE between the CT group and the RT group. The following factors should be considered: The differences in the type of upper limb rehabilitation robots used and the training mode provided by the equipment may lead to biased results. The differences in training duration and training period in each article may indicate the inconsistent total treatment time for stroke patients. At present, the most suitable training time for stroke patients is still not clearly defined, indicating a need for additional research on this aspect. The subgroup analysis of MBI, ARAT, and MAS may face a risk of bias due to the small number of articles and the small sample size.

Currently, there are still some challenges in the clinical application of upper limb rehabilitation robots, such as different initial intervention durations, treatment frequency and cycle, and lack of rigorous multi‐center clinical research. In the future, further research is needed to develop more comprehensive, efficient, and safe multi‐functional rehabilitation robots.

The conclusions of this study should be regarded with caution because of the varied quality of the included studies, which may affect the accuracy of the results. The outcome indicators of the included studies mostly focused on clinical efficacy, and the lack of biochemical indicators inevitably impaired the objectivity of the results.

## 5 Limitations

All the 18 included studies had small sample sizes, which may compromise the robustness of the conclusions due to insufficient data from large cohorts. Second, the quality of the included studies varied significantly, potentially impacting the accuracy of the results. Third, the evaluation method for patient functions is influenced by subjective factors, which may bring bias to the results and undermine the reliability of the conclusions. Therefore, it is essential to interpret these findings with caution. Future research will focus on conducting RCTs with large sample sizes, rigorous design, long‐term follow‐up, and additional neuroimaging indicators to verify the conclusions of this study.

## 6 Conclusion

In conclusion, robot‐assisted training on upper limb rehabilitation can significantly improve the upper limb motor function of stroke patients, reduce muscle tension, and improve the ability of daily living with good safety and compliance.

## Author Contributions

**Tingting Su**: writing–original draft, writing–review and editing. **Mengting Wang**: data curation, writing–review and editing. **Zhouyang Chen**: data curation, writing–review and editing. **Liang Feng**: supervision.

## Disclosure

The authors declare that no funds, grants, or other support were received during the preparation of this manuscript.

## Conflicts of Interest

The authors declare no conflicts of interest.

### Peer Review

The peer review history for this article is available at https://publons.com/publon/10.1002/brb3.70117.

## Supporting information



Appendix S1. The detailed search strategy: PubMed.

## Data Availability

The data that support the findings of this study are available from the corresponding author upon reasonable request.
