# Supplementary material for: Effect of Upper Robot‐Assisted Training on Upper Limb Motor, Daily Life Activities, and Muscular Tone in Patients With Stroke: A Systematic Review and Meta‐Analysis
Source: Brain Behav. 2024 Oct 31;14(11):e70117. doi: 10.1002/brb3.70117 (PMC11527818; doi:10.1002/brb3.70117)
Supplement: Supplementary file 1 — Appendix S1. The detailed search strategy: PubMed. [file BRB3-14-e70117-s001.docx]

**Appendix S1. The detailed search strategy: PubMed**

1. Search: **"Stroke"[Mesh]** Sort by: **Most Recent**

2. Search: **"Robotics"[Mesh]** Sort by: **Most Recent**

3. Search: **((((((((((((((((((((((accident, cerebrovascular) OR (apoplectic stroke)) OR (apoplexia)) OR (apoplexy)) OR (brain accident)) OR (brain attack)) OR (brain blood flow disturbance)) OR (brain vascular accident)) OR (cerebral apoplexia)) OR (cerebral insult)) OR (cerebral stroke)) OR (cerebral vascular accident)) OR (cerebro vascular accident)) OR (cerebrum vascular accident)) OR (cryptogenic stroke)) OR (ischaemic seizure)) OR (ischemic seizure)) OR (stroke)) OR (thrombotic stroke)) OR (cerebrovascular accident)) OR (Apoplexy, Cerebrovascular)) OR (hemiplegic)) OR (paresis)**

4. Search: **(((((((((((((Assistive Robot) OR (Assistive Robots)) OR (robotic therapy)) OR (robot-assisted)) OR (rob**（**1991 to the date of the search**）**otics)) OR (exoskeleton)) OR (robotic aided)) OR (robot assisted)) OR (robotic device)) OR (Robotic, Soft)) OR (Soft Robotics)) OR (Soft Robotic)) OR (Robotics, Soft)**

5. #1 or #3

6. #2 or #4

7. #5 and #6

8. Search: **(randomized controlled trial[pt] OR controlled clinical trial[pt] OR randomized[tiab] OR placebo[tiab] OR drug therapy[sh] OR randomly[tiab] OR trial[tiab] OR groups[tiab]) NOT (animals[mh] NOT humans[mh])**

9. #7 and #8
